# Intron retention as a component of regulated gene expression programs

**DOI:** 10.1007/s00439-017-1791-x

**Published:** 2017-04-08

**Authors:** Aishwarya G. Jacob, Christopher W. J. Smith

**Affiliations:** Department of Biochemistry, University of Cambridge, Tennis Court Road, Cambridge, CB2 1QW UK

## Abstract

Intron retention has long been an exemplar of regulated splicing with case studies of individual events serving as models that provided key mechanistic insights into the process of splicing control. In organisms such as plants and budding yeast, intron retention is well understood as a major mechanism of gene expression regulation. In contrast, in mammalian systems, the extent and functional significance of intron retention have, until recently, remained greatly underappreciated. Technical challenges to the global detection and quantitation of transcripts with retained introns have often led to intron retention being overlooked or dismissed as “noise”. Now, however, with the wealth of information available from high-throughput deep sequencing, combined with focused computational and statistical analyses, we are able to distinguish clear intron retention patterns in various physiological and pathological contexts. Several recent studies have demonstrated intron retention as a central component of gene expression programs during normal development as well as in response to stress and disease. Furthermore, these studies revealed various ways in which intron retention regulates protein isoform production, RNA stability and translation efficiency, and rapid induction of expression via post-transcriptional splicing of retained introns. In this review, we highlight critical findings from these transcriptomic studies and discuss commonalties in the patterns prevalent in intron retention networks at the functional and regulatory levels.

## Introduction

Alternative splicing (AS) is a widespread process, affecting the vast majority of human genes (Barbosa-Morais et al. 2012; Merkin et al. 2012). Many alternative splicing events (ASEs) are regulated to ensure production of appropriate protein isoforms in the correct cellular environments. Numerous examples of the consequences of ASEs upon the function of pairs of protein isoforms have been well documented (Nilsen and Graveley 2010). Alternative cassette exons tend to affect intrinsically disordered protein regions, sites of protein–protein interactions, and sites of post-translational modifications, and programs of AS have the capacity to re-wire protein–protein interaction networks (Buljan et al. 2012; Ellis et al. 2012; Yang et al. 2016). The importance of appropriately regulated AS is underscored by human diseases such as myotonic dystrophy that arise, not from aberrant splicing per se, but from mis-regulation of developmental programs of AS, with clinical symptoms arising from expression of mRNA isoforms at inappropriate stages of development (Cooper et al. 2009). Mis-regulation of alternative splicing is also associated with cancers, and abnormal expression or mutations in splicing factors are known to contribute to tumorigenesis (Anczukow and Krainer 2016).

In addition to the widely appreciated role in production of functionally distinct protein isoforms, many regulated, often highly conserved, ASEs generate mRNA isoforms that are channelled to decay pathways such as nonsense mediated decay (AS-NMD) (Ge and Porse 2014; Hillman et al. 2004; Lareau et al. 2007; Lewis et al. 2003; Weischenfeldt et al. 2012). Such ASEs are frequently referred to as “non-productive” on the basis that one of the RNA isoforms is destined to be degraded rather than translated. However, the “non-productive” label should not be taken to imply lack of functionality. In many cases, the ability to produce a protein-coding or an NMD-targeted mRNA isoform provides an important regulatory function (McGlincy and Smith 2008). For example, the transition between expression of the closely related splicing regulators PTBP1 and PTBP2, which is important during neuronal differentiation, is effected by an AS-NMD event in the PTBP2 pre-mRNA that is antagonistically regulated by PTBP1 and RBFOX proteins (Boutz et al. 2007; Jangi et al. 2014; Makeyev et al. 2007). Indeed, the presence of such AS-NMD events within the pre-mRNAs of splicing regulatory proteins allows for auto-regulation and cross-regulation between families of related proteins, as well as control by “master” RBPs, which in turn helps to create robust post-transcriptional regulatory networks (Jangi and Sharp 2014).

AS is commonly classified into seven types of simple binary events: cassette exons, mutually exclusive exons, alternative 5′ splice sites, alternative 3′ splice sites, intron retention (IR), alternative 3′ terminal exons, and alternative 5′ exons. In addition, many complex ASEs involve combinations of these simple events (Vaquero-Garcia et al. 2016). Of the classes of ASE, IR has probably received the least attention in humans and other mammals, at least until recently. This may have resulted in part because of the difficulty in determining unequivocally that an apparent IR event derives neither from genomic DNA nor from RNA processing intermediates. In contrast to its relatively neglected role in human gene-expression, IR is the most common type of ASE in plants, fungi, and unicellular eukaryotes, and has consequently long been appreciated as an important regulatory mechanism by researchers using these model organisms (Pleiss et al. 2007; Syed et al. 2012). Regulated splicing of intron 3 of the *Drosophila* P-element transposase was one of the earliest examples of cell-type specific AS regulation with clear-cut consequences for the activity of the encoded protein (Rio et al. 1986). Splicing of P-element intron 3 in germ-cells produces the full length transposase, while retention of intron 3 in somatic cells gives rise to a shorter DNA binding protein that lacks transposase activity and acts as an antagonist of the full-length protein. The P-element transposase also showed how IR can be regulated in a cell-type specific manner via repressors of intron 3 splicing in somatic cells [e.g., (Adams et al. 1997; Horan et al. 2015; Labourier et al. 2001)]. In view of this long acknowledged role in many other organisms, the recent emergence of the varied roles of IR in humans and other mammals should come as no surprise (Ge and Porse 2014; Wong et al. 2016). Moreover, in addition to physiologically regulated events, aberrant IR can result from mutations in splice sites or regulatory sequences. Disease-associated mutations in splice sites are most frequently associated with exon skipping (Berget 1995), but in many cases, mutation driven IR can be pathological (Wong et al. 2016). For example, IR has been identified as a common cause of tumor-suppressor inactivation in cancers (Jung et al. 2015).

**Fig. 1:**
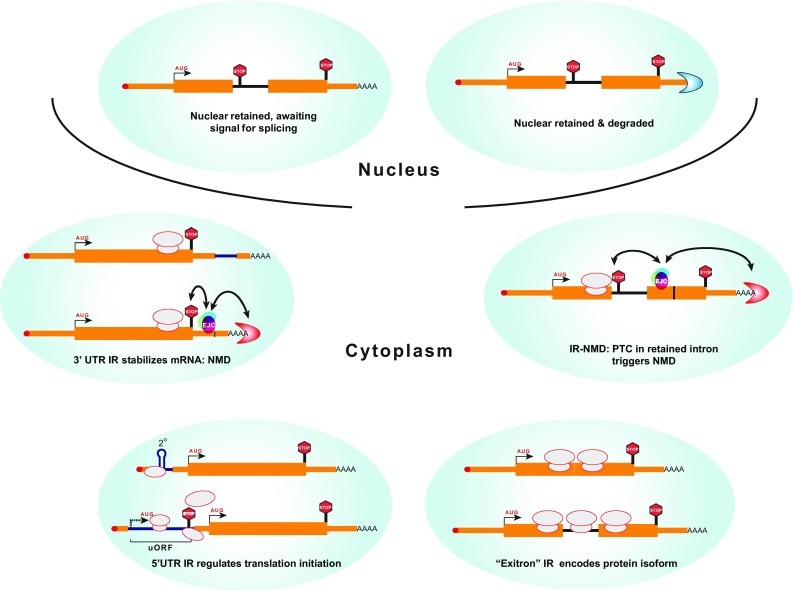
Functionally diverse consequences of intron retention. Schematic illustration of functional consequences of IR. In all cases, the *thin black line* represents the retained intron. The remainder of the transcripts is shown in *orange*, with the main ORF defined by the non-IR isoform shown wider, and the UTRs shown as *thinner orange blocks*. The 5′ cap is shown as a *red circle*. IR can lead to nuclear retention associated with nuclear degradation involving the exosome. Alternatively, nuclear retained IR-RNAs can be stable, awaiting a signal for post-transcriptional splicing. Cytoplasmic IR-RNAs with IR in the main ORF can be targeted by the NMD machinery, due to insertion of PTCs, or they can encode full length protein isoforms. IR within the 5′ UTR has the potential to regulate translation initiation in a number of ways, most commonly repressing translation of the main ORF via the action of upstream ORFs (uORFs), or via secondary structure and longer 5′ UTRs, which can render the mRNA sensitive to inhibition by eIF4EBPs [e.g., (Tahmasebi et al. 2016)]. Conversely, IR in the 3′ UTR can up-regulate stability, because splicing of introns in the 3′ UTR can lead to NMD (Sun et al. 2010). In addition, IR in the 3′ UTR could introduce regulatory elements bound by proteins or miRNAs, which could regulate mRNA stability and translation in various ways (Thiele et al. 2006)

Intron retention is most often associated with down-regulation of gene expression via NMD (IR-NMD) (Ge and Porse 2014) primarily because retained intron sequences that interrupt the main open reading frame (ORF) of the mRNA usually lead to introduction of premature termination codons (PTCs). However, this is by no means the only consequence. The fate of an mRNA with one or more IR events depends upon a number of factors, including the location of the IR event within the transcript (Fig. 1):

- Nuclear retention and degradation.
- Nuclear retention and storage awaiting signal-induced splicing.
- IR in the 5′ UTR can insert an upstream ORF (uORF) or other structural features that can activate or repress translational initiation efficiency.
- IR in the main ORF can result in PTCs leading to IR-NMD, or possibly production of truncated proteins.
- IR in the main ORF can maintain reading frame allowing production of pairs of protein isoforms.
- If the intron is more than ~55 nt into the 3′ UTR, where splicing would lead to NMD, IR can stabilize the RNA by avoiding NMD.
- IR in the 3′ UTR can introduce *cis*-elements that affect the stability or translational efficiency of the mRNA.

Here, we review progress in understanding the contributions of regulated IR in mammalian cells and highlight examples of its various roles in gene expression modulation. In particular, we focus on recent transcriptome-wide analyses, including those of developmentally regulated gene expression programs, where IR plays important roles.

## The challenges of detecting and defining intron retention events

**Fig. 2:**
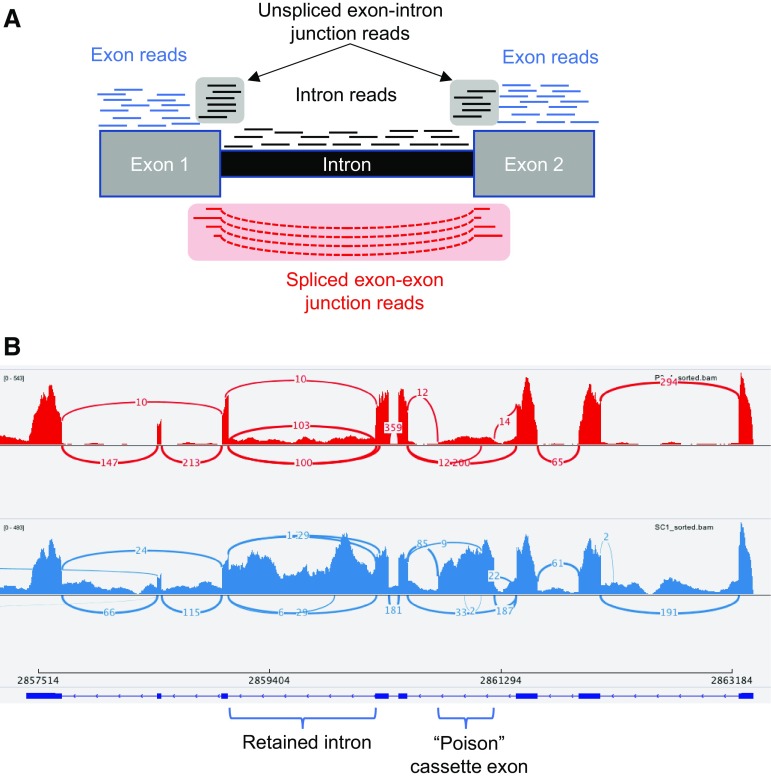
Intron retention profiling by mRNA-Seq. **a** Schematic diagram showing distribution of sequence reads informative for intron retention. Percent intron retention can be calculated from the ratio of unspliced exon–intron junction reads to total junction reads (unspliced exon–intron and spliced exon–exon), or from the read density across the intron compared to adjacent exons. Uniform read density across the intron rules out alternative processing events. **b** Example of mRNA-Seq data from rat primary aorta smooth muscle cells (unpublished data). Differentiated, *blue lower panel*; proliferative, *red upper panel*. The Sashimi plot, generated from the Integrative Genomics Viewer (Robinson et al. 2011), shows the Srsf7 gene. In differentiated cells, there is substantial IR in intron 6, as well as inclusion of the known “poison” cassette exon between protein coding exons 3 and 4 (Lareau et al. 2007)

IR is fundamentally different from other simple ASEs in that the sequence of the products of IR is identical to that of genomic DNA and pre-mRNA (at least in the area of the IR event). This means that precautions need to be taken to ensure that observed IR products are indeed derived from processed RNA. This involves the use of routine controls, such as omission of reverse-transcriptase to rule out genomic DNA as the source template, and the use of oligo dT selection of RNA for priming of cDNA synthesis to ensure that poly-adenylated RNA is being analyzed. Use of cytoplasmic polyA+ RNA can help to reduce the signal from nascent RNA, but at the expense of missing functionally important nuclear-retained RNA species (see in the following). Many IR products are much longer than their spliced counterparts, meaning that it is not always possible to obtain single-reads that unambiguously cover both exon–intron junctions as well as the entire intron. Nevertheless, a range of approaches have been used to identify and profile intron retention using next generation sequencing (NGS) (Braunschweig et al. 2014; Marquez et al. 2015; Pimentel et al. 2016; Wong et al. 2013). These involve a combination of quantitating reads across unspliced exon–intron junctions and spliced exon–exon junctions as well as comparison of reads within introns to those mapping to adjacent exons (Fig. 2), allowing IR to be measured as “percent intron retention” (PIR). The use of a combination of approaches is necessary to unequivocally determine the occurrence of IR, and to rule out other processes, such as use of alternative 5′ or 3′ splice sites or polyA signals that can lead to inclusion of parts of annotated introns into the processed RNA.

Another challenge with IR is that, while a static snapshot of the transcriptome can reveal for other types of events that a splicing decision has been made—for example, to include or skip a cassette exon—the observation of a retained intron in polyadenylated RNA is ambiguous. It could either represent a mature fully processed RNA; an intermediate that accumulates prior to a slow rate-limiting splicing step; or one that is deliberately stalled awaiting a decision on whether or not to be spliced post-transcriptionally. Indeed, it is now recognized that a subset of IR events are present in RNAs that are “detained” in the nucleus awaiting a specific signal to be spliced (Boutz et al. 2015; Mauger et al. 2016). The observation of a retained intron in cytoplasmic polyA+ RNA gives some confidence that the RNA is indeed an end-product. However, even this confidence is challenged by the observation that IR RNAs in the cytoplasm of platelets (which have no nucleus) can be spliced in response to activating signals (Denis et al. 2005).

## Global profiling of IR

A number of transcriptome-wide analyses of IR have recently been published, many of them focusing on specific systems of cellular differentiation or responses to stress. Braunschweig et al. carried out an extensive and deep quantitative survey of IR in PolyA+ mRNA across 40 human and mouse tissues, providing a range of insights into the prevalence, biological roles, and regulation of IR (Braunschweig et al. 2014). Around half of all introns (in 77% of genes) were observed to have a PIR >10% in at least one tissue, with 8–9% of introns (35% of genes) having a PIR of >50% in at least one sample. IR was highest in neurons and immune cells and lowest in ES and muscle cells. Comparison across multiple species showed that tissue-specific IR was most conserved in neurons, as had been observed for other classes of ASE (Barbosa-Morais et al. 2012; Merkin et al. 2012). Compared with constitutively spliced introns, IR was enriched in untranslated regions (UTRs) and non-coding RNAs, depleted in protein coding regions, and tended to be higher towards the 3′ end of RNAs. Overall, levels of IR were higher in the nucleus than the cytoplasm, consistent with either nuclear retention or cytoplasmic NMD of IR RNAs, in agreement with a number of other reports associating IR with NMD or nuclear retention (Boutz et al. 2015; Edwards et al. 2016; Llorian et al. 2016; Mauger et al. 2016; Pimentel et al. 2016; Shalgi et al. 2014; Wong et al. 2013; Yap et al. 2012).

Retained introns could be classified into three groups, varying by their characteristic PIR, GC-content, intron length, evolutionary history, and their effects upon the ORF (Braunschweig et al. 2014). The most abundant group (referred to as Class A) show low PIR, intermediate GC content, and intron length and are derived from ancestral introns. Class B IR events occur within annotated exons, have a high PIR and GC-content, are short, and appear to be derived via intronization from ancestral exons (Braunschweig et al. 2014; Marquez et al. 2015). Class C are characterized by being adjacent to annotated cassette exons, and show intermediate PIR, with low GC% and long introns. The Class C events have been observed in a number of other investigations (see in the following) and might be associated with the fact that although most splicing is co-transcriptional (Tilgner et al. 2012), regulated ASEs are often spliced more slowly post-transcriptionally (Pandya-Jones et al. 2013; Pandya-Jones and Black 2009) and, in some cases, remote from the site of transcription (Vargas et al. 2011). In agreement with previous characterizations of IR (Sakabe and de Souza 2007), all three classes of IR had weaker splice sites than constitutive introns, with the class B events having particularly weak sites, consistent with their higher PIR levels.

## Protein-coding IR

An independent global analysis of IR, based on AS profiling in *Arabidopsis thalania* (Marquez et al. 2012), also converged on the protein-coding Class B events as an interesting, functionally, and evolutionarily distinct subset of IR in plants and humans (Marquez et al. 2015). In *A. thalania,* 11% of IR events occurred within annotated exons, with both mRNA isoforms being translated in the cytoplasm to produce distinct protein isoforms, and with the longer IR form predominating. Marquez et al. termed these “exitrons” (exonic introns) in recognition of their dual nature and also of the ambiguous terminology that has previously been applied to such events. The majority of exitrons, with length in multiples of 3 nt, maintain the same reading frame upon retention or splicing, although some alter the reading frame on the downstream side. Exitrons often encode intrinsically disordered protein regions and are enriched for short linear peptide motifs and residues subject to various post-translational modifications (Marquez et al. 2015), similar to cassette exons (Buljan et al. 2012; Ellis et al. 2012). A number of interesting examples where modification of function is apparent include the translation initiation factor and ATP-dependent RNA helicase, eIF4A1. In both *Arabidopsis* and humans, a shorter exitron-spliced form lacks both the ATP-binding motif and two regulatory phosphorylation sites that are present in the full-length IR isoform. Other mammalian examples include events in the DNA-binding transcription and replication factor CIZ1, where exitron splicing reduces nuclear matrix localization and has been associated with Alzheimer’s disease (Dahmcke et al. 2008), FOSB where altered splicing is associated with breast cancer (Marquez et al. 2015), and the nuclear export factor NXF1. Retention of *NXF1* intron 10 allows its mRNA to be bound by NXF1 protein at a conserved transport element (CTE), possibly as part of a feedback loop. This allows for the *NXF1* IR isoform to be transported to the cytosol and translated into a C-terminal truncated sNXF1 protein that serves as a cofactor to its full-length counterpart. sNXF1 has recently been shown to be expressed in hippocampal and cortical neurons, localizing in cytoplasmic granules suggestive of functionality in the cytosolic export of the other intron containing mRNA (Li et al. 2016).

Phylogenetic comparisons suggest that the exitron class of IR event is derived by a process of intronization of ancestral exonic sequences, which at some point acquired splice sites (Braunschweig et al. 2014; Marquez et al. 2015). In support of this, cross-species comparisons identified a number of cases where the orthologous sequence is contained within a separate exon. The high basal PIR level of the exitron events would be consistent with the continued importance of the ancestral full-length protein isoform, with a more newly acquired regulatory function being provided by the shorter spliced form (Marquez et al. 2015). Although individual cases of IR leading to pairs of functional protein isoforms had previously been reported, these two systematic analyses now reveal that a significant minority of IR events contribute to protein isoform diversity in a very similar way to cassette exons (Braunschweig et al. 2014; Marquez et al. 2015).

## IR and translation

Despite the overwhelming evidence of the contribution of ASEs to generation of transcriptomic complexity, mass spectrometric analyses have not always captured the full diversity of protein isoforms [e.g., (Ezkurdia et al. 2015)], possibly due to limitations in sensitivity and coverage. A number of groups have recently used complementary approaches to monitor the extent to which AS RNA isoforms associate with ribosomes and to ask whether alternative isoforms are differentially translated. Although not focused upon IR, all these reports found that IR events were detectable in cytoplasmic fractions and to variable extents engaged with ribosomes (Floor and Doudna 2016; Shalgi et al. 2014; Sterne-Weiler et al. 2013; Weatheritt et al. 2016). In addition to productive translation of protein-coding IR isoforms (Marquez et al. 2015), ribosome-association would be consistent with RNAs being degraded by NMD, which relies upon a pioneer round of translation (Maquat et al. 2010), and with ribosome association with uORFs. High-resolution fractionation of polysomes into different size classes, from 1 to 8 or more ribosomes, showed that most IR events were present in a cluster of poorly translated transcripts (Floor and Doudna 2016). This is consistent with IR leading to down-regulation of protein expression via NMD. In contrast, a small number of IR containing transcripts were enriched in larger polysomes. It seems likely that these transcripts would be enriched for protein-coding “exitron” events (Floor and Doudna 2016).

As an alternative to polysome profiling, Weatheritt et al. combined ribosome “footprinting” with mRNA-Seq to assess ribosomal engagement by mRNA isoforms (Weatheritt et al. 2016). This revealed that IR was under-represented on ribosomes compared to whole cell RNA, but was comparable between cytosolic mRNA and ribosomes. Here also, IR events were most enriched among the lowest expressed RNAs, consistent with ribosome engagement as a precursor to NMD. However, a smaller group of ribosomally engaged IR events was enriched in the 5′ UTRs of highly expressed mRNAs encoding a range of essential housekeeping proteins involved in cell cycle, translation, DNA repair, and transcription (Weatheritt et al. 2016). Compared to all 5′ UTR IR events, these ribosomally engaged IR events were highly enriched for annotated uORFs, consistent with their identification by 5′ UTR ribosomal footprint reads. This indicates that IR can lead to translational regulation by modifying the 5′ UTR, as observed for other types of ASE (Floor and Doudna 2016; Sterne-Weiler et al. 2013). Because these observations were made with ~30 nt ribosome-protected reads, it is not possible to directly infer whether uORF ribosome occupancy is linked to translational up- or down-regulation of the main ORF on the same RNA. However, using data from different stages of the cell cycle, clear correlations were observed between PIR of the 5′ UTR introns and ribosome occupancy of main ORFs. For example, higher PIR in the 5′ UTR of CDC20 during the G1 phase of the cell cycle correlated with higher ribosome occupancy of the main ORF—an example of IR leading to up-regulation of gene expression. This class of 5´ UTR IR events was also shorter than most IR introns and appears to have arisen by intronization (Weatheritt et al. 2016). They, therefore, resemble the exitron events in many respects, with the exception that they do not occur within the main ORF.

A recently evolved 5′ UTR IR event in the mRNA for the murine YY2 transcription factor illustrates both how such an IR event can be important in translational control of development, and also how the IR event itself can be regulated by RNA binding proteins (Tahmasebi et al. 2016). The IR mRNA isoform of *YY2* is translationally down-regulated via the EIF4E binding proteins (EIF4EBP 1 and 2). Here, the repressive effect of IR upon translation is not via uORFs, but appears to be related to the resultant longer, more structured 5′ UTR that confers EIF4EBP sensitivity. Splicing of the intron removes 117 nt from the 5′ UTR and the shorter *YY2* isoform is not subject to EIF4EBP-mediated repression. This leads to the upregulation of YY2 and differentiation into the cardiovascular lineage (Tahmasebi et al. 2016). Interestingly, the IR in *YY2* is promoted by PTBP1, a well-known splicing repressor (Keppetipola et al. 2012), and decreases during differentiation concordant with PTBP1 expression, presenting with the lowest levels in terminally differentiated tissues such as the heart. This PTBP-mediated IR regulation of YY2 is similar in several respects to the regulation in *Drosophila* of Male Specific Lethal-2 (MSL2) expression by the RNA binding protein sex-lethal (SXL). SXL protein binds to the *MSL2* 5′ UTR inhibiting the splicing of a resident intron (Merendino et al. 1999). The *MSL2* transcript with Sxl bound to the long 5′ UTR is both inhibited for translation (Bashaw and Baker 1997; Gebauer et al. 1998; Kelley et al. 1997) and for nuclear export (Graindorge et al. 2013).

Overall, these preceding examples illustrate how IR can influence ribosomal association, either as a precursor to NMD or by regulating translation initiation through uORFs or other sequences that can either repress or activate translation initiation.

## IR events represent asymmetrically co-regulated components of AS programs

A number of recent reports have documented extensive changes in IR as part of developmentally regulated gene expression programs in haematopoietic cells (Cho et al. 2014; Edwards et al. 2016; Ni et al. 2016; Pimentel et al. 2016; Wong et al. 2013), neurons (Braunschweig et al. 2014; Yap et al. 2012), breast epithelial cells (Gascard et al. 2015), and smooth muscle cells (Llorian et al. 2016). In addition, co-regulated programs of IR have been observed in response to heat shock (Shalgi et al. 2014), neuronal activation (Mauger et al. 2016), inhibition of CLK kinases (Boutz et al. 2015), DNA damage (Boutz et al. 2015), tumor hypoxia (Memon et al. 2016), and in various cancers (Dvinge and Bradley 2015; Jung et al. 2015). Comparison of these investigations reveals both common and program-specific features of IR-mediated regulation.

A consistent feature of co-ordinated IR programs is that the vast majority of IR events change in the same direction. This asymmetric response is apparent during cellular differentiation, cell activation, stress or cancer, and stands in stark contrast to other types of ASE, such as cassette exons, which generally show similar numbers of events with increased inclusion or skipping. In the developmentally regulated programs, the common tendency is for increased intron retention in the more differentiated or quiescent cell state (Braunschweig et al. 2014; Cho et al. 2014; Edwards et al. 2016; Gascard et al. 2015; Llorian et al. 2016; Ni et al. 2016; Pimentel et al. 2014, 2016; Wong et al. 2013). However, a high PIR state is not necessarily an indication of terminal differentiation. Activation of CD4+ T cells (Ni et al. 2016) and de-differentiation of smooth muscle cells toward proliferative states (Llorian et al. 2016) trigger dramatic decreases in IR. Other differentiation programs involve more complex patterns of IR. For instance, the final four stages of erythropoiesis involve progressively increasing IR before achieving a terminal erythrocytic stage that has lower IR compared to the precursor cells common to both megakaryocytes and erythrocytes (MEP cells) (Edwards et al. 2016; Pimentel et al. 2016). This highly dynamic pattern is further complicated by the presence of subgroups of IR events that exhibit differential or opposing regulation between the different stages of erythroblast differentiation (Edwards et al. 2016; Pimentel et al. 2016). Even neurons that are typically characterized by increasing IR during differentiation exhibit a small class of terminal introns whose retention by PTBP1-mediated repression is relieved upon PTBP1 reduction in differentiating neurons (Yap et al. 2012). In general, however, it is interesting to note that differentiation programs directing toward terminal or more permanent post-mitotic states frequently tend to exhibit increased IR patterns, while more plastic cell types whose functional maturation involves transition to proliferative states show decreased IR incidence.

On the other hand, in cancers, IR tends to be higher than in normal adjacent control tissue (Dvinge and Bradley 2015) and is often a mechanism for downregulating tumor suppressor expression (Jung et al. 2015). Using data from the Cancer Genome Atlas (TCGA), Dvinge and Bradley explored the contribution of IR to the transcriptomes of 16 cancer types (Dvinge and Bradley 2015). Once again, IR behaved asymmetrically and the lack of observed mis-pairing of constitutive splice sites argued against a contribution of “splicing noise”. Exceptions to the general rule are breast tumors, where the healthy tissue that shows significant upregulation of IR during normal physiological development (Gascard et al. 2015), is the outlier with high levels of IR compared to the matched tumors (Dvinge and Bradley 2015). In the case of normal breast tissue, IR bears distinct signatures between the luminal and myoepithelial cells with luminal cells expressing over seven times the number of IR events as myoepithelial cells (Gascard et al. 2015). Hence, it would be interesting to compare IR in basal and luminal tumors with the corresponding normal cell type. Another example includes myelodysplasia where intron retention is higher in wild type or healthy control than in SF3B1 mutant cells (Dolatshad et al. 2016). The tendency for higher IR in cancer cells might be related to the apparent vulnerability of the spliceosome in *MYC* transformed cells, as indicated by synthetic lethality between *MYC* transformation and knockdown of core splicing factors (Hsu et al. 2015). Partial inhibition of splicing in *MYC* transformed cells leads to global increases in IR, interpreted as being due to the extra load imposed on the splicing machinery by the overall increased transcription in response to MYC transformation (Hsu et al. 2015). In contrast, in many differentiated cells where IR prevails, levels of core splicing factors are lower than in their less differentiated counterparts (Gascard et al. 2015; Llorian et al. 2016; Pimentel et al. 2014, 2016; Wong et al. 2013). Hence, regulated IR events might in general be especially vulnerable to limiting spliceosome availability resulting either from reduced levels of the components in quiescent differentiated cells or competition in highly transcriptionally activated transformed cells.

## Targets of regulated IR events in various AS programs

One consistent pattern that has emerged across many biological contexts is that regulatory IR particularly affects spliceosome components, splicing factors, and other post-transcriptional regulators (Boutz et al. 2015; Dvinge and Bradley 2015; Edwards et al. 2016; Gascard et al. 2015; Llorian et al. 2016; Memon et al. 2016; Pimentel et al. 2016; Shalgi et al. 2014). The splicing factors include core components of U1 (Snrnp70) and U2 snRNPs (Sf3b1, Snrpa1) as well as regulatory factors such as SR proteins (Srsf1, 2, 3, 5, 7). In many cases, these IR events are associated with alternative “NMD-switch” cassette exons that can lead to generation of PTCs upon inclusion (e.g., SR proteins, Snrp70) or upon skipping (e.g., Clk1, Clk4, and Snrpa1) (Boutz et al. 2015; Lareau et al. 2007; Llorian et al. 2016; Pimentel et al. 2014, 2016), and so typify the Class C IR events described in (Braunschweig et al. 2014). They also mostly appear to be retained in the nucleus, and some of them represent stable intermediates that can be post-transcriptionally spliced in response to signalling (Boutz et al. 2015; Mauger et al. 2016). The IR events in splicing factor pre-mRNAs, along with other non-productive alternative splicing patterns (Llorian et al. 2016; Pimentel et al. 2014, 2016) all act to down-regulate expression of splicing factors in a coordinated fashion. This suggests a global regulatory network in which numerous splicing factors and other post-transcriptional regulators are set to a low-expression state by IR in differentiated or quiescent cells.

Intron retention events also affect expression of proteins with cell type-specific functions. One prominent example for the impact of IR on cellular function lies in granulocyte biology. These cells are marked by an unusual multi-lobed nuclear morphology which, being more deformable than conventional spheroid nuclei, enables them to transit the endothelial lining of blood vessels and move through tissue interstitial spaces. Interestingly, genes encoding proteins associated with the nuclear periphery or nuclear lamina represent up to 25% of the IR events with increased PIR during differentiation from pro-myelocytes to granulocytes (Wong et al. 2013). In most cases, the IR event led to NMD of the resident transcripts and downregulation of gene expression. A similar set of genes was regulated by IR in mouse and humans although not always via the orthologous introns. A striking example was LaminB1 (Lmnb1), a constituent of the nuclear lamina associated with the inner nuclear membrane. Here, retention of introns 5–10 was up-regulated more than 100-fold, while the mRNA was down-regulated by 100-fold during differentiation. Enforced expression of Lmnb1, not subject to down-regulation by IR, led to reduced numbers of circulating granulocytes with increased nuclear volume and altered nuclear morphology. The IR-NMD mediated down-regulation of the genes associated with nuclear peripheral structure, therefore, appears to be important for the proper development of the mature granulocyte phenotype (Wong et al. 2013). In differentiating erythroid cells, IR is seen to affect a number of genes with important cell specific roles, including in haem biosynthesis and iron homeostasis (Edwards et al. 2016; Pimentel et al. 2016). On the other hand, T-cell activation is accompanied by decreased IR and increased mRNA levels for proteasome components, which are important for proliferation and cytokine release (Ni et al. 2016).

Differentiation of glutamatergic neurons from mES cells features a program of progressively increasing IR that generally correlated with lower transcript levels and affected genes associated with DNA replication and pluripotency (Braunschweig et al. 2014). A smaller number of genes with decreasing IR were associated with neuron specific functions. Similarly, in mouse neuroblastoma cells, a small set of 3′ terminal introns were identified that are retained under the influence of the splicing repressor PTBP1. The IR RNAs were not subject to NMD, but were retained and turned over by the exosome complex in the nucleus (Yap et al. 2012). The affected genes were enriched for proteins with neuronal post-synaptic functions. Interestingly, variations in IR have also been observed between different regions of the mouse brain. Several genes especially those involved in Glutamate receptor signalling pathway such as *Grm1* (mGluR1) and *Grm5* (mGluR5) were differentially regulated by IR between the cerebellum and the cerebrum highlighting a role for IR in synaptic plasticity (Martin et al. 2016). IR, therefore, leads to down-regulation of proteins essential for neuronal function before the cells are differentiated.

The preceding examples show how regulated IR, coupled to either cytoplasmic NMD or nuclear degradation, can be used for tissue-specific fine-tuning of the transcriptome. Further refinements to the use of IR-NMD can be added by coupling with temporally or spatially regulated translation. For example, Robo3, a gene essential for axon guidance in the spinal cord during embryonic development, uses IR-NMD coupled with translational control to precisely modulate the levels and spatial expression patterns of its two antagonistic isoforms Robo 3.1 and 3.2 (Chen et al. 2008). Robo3.2, whose expression is only required in post-crossing neurons, retains intron 26 (of 27) which introduces a PTC and makes Robo3.2 an NMD target. Prior to midline crossing, Robo3.2 mRNAs are confined to the cell bodies and translationally repressed. As a result, while Robo3.2 transcripts are detectable, its encoded protein is not. Once the axons cross the ventral midline, Robo3.1 protein levels drop and Robo3.2 mRNA is transported to the axons where it is locally translated. This, in turn, triggers NMD leading to a short pulse of low Robo3.2 expression at the appropriate location (Colak et al. 2013; Ge and Porse 2014). Consistent with the importance of the limiting Robo3.2 expression by IR-NMD, mouse embryos with conditional knockout of the NMD factor Upf2, showed disrupted axonal trajectories.

## Heat shock induced IR

Eukaryotic cells respond to various stresses by concerted responses at all levels of gene expression from transcription to translation, including RNA processing (Biamonti and Caceres 2009). The response to heat shock involves down-regulation of global gene expression with maintained or enhanced expression of protective proteins such as chaperones. Previous work had pointed to the importance of the splicing regulator SRSF10 (formerly SRp38) in this response (Shi and Manley 2007), and also the accumulation of various splicing factors along with heat shock transcription factor 1, HSF1 (Biamonti and Vourc’h 2010) and Bromodomain containing protein BRD4 (Hussong et al. 2017) in nuclear stress bodies. Transcriptional profiling of mouse 3T3 cells subjected to mild or severe heat shock revealed the full extent of the splicing response (Shalgi et al. 2014). As in other regulated programs, most types of AS showed similar numbers of events changing in each direction, but the most prominent response was an increase in IR. Over half of IR events changed significantly and of these 74% showed increased retention. Moreover, multiple introns were affected in individual genes, suggesting a gene-level rather than an individual intron-level response. Importantly, the IR RNAs, were neither exported to the cytosol nor translated but were stably retained in the nucleus, potentially as a pool of precursors that can be readily spliced and activated for recovery of normal gene expression post-stress. Genes affected by IR were enriched for functions associated with splicing, nuclear pore and tRNA synthetases, consistent with amplification of the widespread downregulation of gene expression in response to heat stress.

In contrast, a set of 583 genes, including those with functions required for the immediate response to heat shock such as protein-folding, were “unaffected” by IR. Newly synthesized RNA from these genes appeared to be spliced co-transcriptionally with high efficiency as evidenced by their loss from chromatin-associated sub-nuclear fractions in heat-shocked cells compared to controls. Indeed, the unaffected RNAs were actually spliced more efficiently under heat shock, perhaps in association with recruitment to nuclear stress bodies (Biamonti and Vourc’h 2010). However, IR appeared to be concentrated within the post-transcriptionally spliced RNAs both in heat shock as well as normal conditions (Shalgi et al. 2014). Overall, the heat shock IR response appears to focus upon subsets of genes that are already distinguished by the spatial and temporal relationship of transcription and RNA processing.

## “Detained introns” and post-transcriptional splicing

In contrast to the “gene-level” IR observed in heat shock, Boutz et al. described a distinct set of “detained introns” (DI), defined as unspliced introns in otherwise fully spliced polyA+ mRNA from mouse ES cells (Boutz et al. 2015). A primary consequence of detained introns is nuclear retention, with the RNA either eventually being spliced to completion and exported, or turned over in the nucleus. In many cases, detained intron events are adjacent to NMD-switch exons and the high PIR state is associated with exon skipping, whereas post-transcriptional splicing involves exon inclusion. For instance, the Clk1 and Clk4 kinases that phosphorylate critical splicing regulatory SR proteins (Fu and Ares 2014) are themselves subject to regulation by detained introns. Clk1 mRNA retains introns flanking a cassette exon that can be spliced post-transcriptionally in response to osmotic shock or, in a feedback loop upon pharmacological inhibition of its own kinase activity (Ninomiya et al. 2011). In mESCs, Clk inhibition induced altered intron detention and post-transcriptional splicing of 10% of the total of ~3000 observed detained intron events, with 4% showing decreased and 6% increased retention. Prominent regulated targets included Clk1 and 4, as expected, but also several of their substrate Ser-Arg rich proteins including Srsf3, 5, and 7 (Boutz et al. 2015) making these a functionally coherent group of coregulated transcripts. In each case, splicing of the detained introns upon Clk inhibition caused inclusion of the adjacent cassette exon, although the functional outcomes observed were opposite for the Clk kinases and their substrates. While Clk1 and 4 increased inclusion of a coding exon (NMD-skip event) upon DI splicing, Srsf3, 5 and 7 spliced in one of the well-characterized “poison” PTC-containing exons (Lareau et al. 2007) upon their DI removal. The Clk1 and 4 IR events were also observed to respond to endoplasmic reticulum (ER) stress, but not starvation stress, by increased post-transcriptional splicing in intestinal organoids (Tsalikis et al. 2016). This was despite the fact that starvation stress had much more widespread IR effects than ER stress, showing specificity in the response of detained intron events to different stimuli. Further specificity was evident from the response to DNA damage in which a distinct set of DI events were regulated (Boutz et al. 2015). The effect of post-transcriptional splicing of the detained introns in Clk1 and 4 is to switch from a paused OFF state to an ON state. However, for the SR proteins, the delayed splicing acts to confirm the initially transient OFF state by channeling the spliced product to NMD. In this capacity, they represent intricate regulatory mechanisms that serve to toggle specific gene expression states in response to external cues. It is not clear whether the detained intron events associated with alternative cassette exons are always committed to exon inclusion upon activation of splicing. It would be particularly interesting if the detained RNAs retain flexibility so that different stimuli could promote either exon skipping or inclusion.

Post-transcriptional splicing of nuclear-detained introns also occurs in mouse neurons in response to GABA_A_ receptor activation, which increases neuronal network activity (Mauger et al. 2016). RNA-Seq of polyA+ RNA from mouse neocortex and from cultured neurons identified ~10,000 IR events, the majority of which were in stable RNAs. A significant sub-set was shown to alter their PIR substantially as early as 15 min after GABA_A_ receptor activation in neurons. Pre-treatment with the transcription inhibitor DRB ruled out any contribution of de novo transcription, and the reciprocal increased levels of spliced products and decreased PIR for 221 introns, strongly supported the conclusion that neuronal activation led to post-transcriptional splicing of a subset of IR events. Moreover, the higher levels of spliced mRNAs were associated with ribosomes in the cytoplasm, indicating that the activation of splicing rapidly fed through to new protein synthesis. The regulated IR events tended to affect a single intron in each gene, and were associated with long pre-mRNAs, which are themselves characteristic of neurons (Gabel et al. 2015; Sibley et al. 2015). Rapid gene expression responses are essential for neuronal plasticity. Consistent with this, immediate early response genes tend to be very short. The presence of a pool of nuclear pre-mRNAs with a single unspliced intron provides an alternative mechanism for the very rapid induction of expression of long genes for which de novo transcription would take several hours to provide any response (Mauger et al. 2016).

A striking example of delayed post-transcriptional splicing is provided by the induction of IL1β and tissue factor (TF) expression in platelets. Unspliced IL1β and TF pre-mRNAs are transcribed in megakaryocytes and persist through to anucleate platelets, where they can be spliced upon platelet activation (Denis et al. 2005; Schwertz et al. 2006; Shashkin et al. 2008). For both IL1β and TF, unspliced intron-containing pre-mRNA was rapidly converted to spliced mRNA upon activation by various agonists, and active protein produced. In the case of TF, the activation pathway involved Clk1 kinase, as indicated by the use of Clk inhibitors (Schwertz et al. 2006). These examples show how splicing can be delayed to allow rapid switching on in response to appropriate signals, even in cells that are no longer transcriptionally active. Presumably the un-spliced RNAs are translationally repressed before activation to avoid degradation by NMD. The platelet examples raise the question of how many other RNAs might be post-transcriptionally spliced in the cytoplasm. Indeed, extensive IR was observed in megakaryocytes, the precursors to the anucleate platelets, and in orthoblastic erythroblasts the precursors to anucleate erythrocytes (Edwards et al. 2016; Pimentel et al. 2016). It is possible that some of these IR transcripts might also be spliced in the mature platelets or possibly even erythrocytes (Edwards et al. 2016). It has been argued that regulated cytoplasmic splicing might occur in other specialized cell types too, for example in neuronal dendrites where both spliceosome components and intronic RNA sequences have been observed [discussed in (Buckley et al. 2014)]. However, the evidence for cytoplasmic splicing is less clear-cut in this case; at least some of the events referred to as intron retention actually involve use of previously unannotated 3′ splice sites (Bell et al. 2010), leading to “retention” of sequences previously annotated as intronic only, but not conforming to a strict definition of IR.

## Mechanisms of IR regulation

IR resulting from mutation of splice sites is a diagnostic test for whether splicing complexes initially assemble across an intron (intron definition). More commonly in human genes, splice site mutations result in exon skipping reflecting initial recognition of splice site pairs across an exon (exon definition) which would be followed later on by cross-intron spliceosome assembly (Berget 1995). Whether pairs of splice sites are initially defined and paired across introns or exons depends upon a number of features, including exon and intron length and also their relative GC content (Amit et al. 2012; Berget 1995). Shorter introns with higher GC content tend to be initially recognized as a unit (intron definition), whereas short exons flanked by longer introns with lower GC content tend to be recognized by initial exon definition. Indeed, tumor-associated introns retained as a result of allele-specific sequence variants at the last base of the exon showed high intronic GC-content consistent with the defined characteristics for intron definition (Jung et al. 2015).

It seems reasonable to expect that physiological IR in the absence of *cis* mutations will also occur predominantly where intron definition operates. Indeed, many investigations of mammalian IR have noted common shared features, including short intron length and higher GC content, which are also associated with intron definition (Braunschweig et al. 2014; Dvinge and Bradley 2015; Llorian et al. 2016; Marquez et al. 2015; Pimentel et al. 2016; Sakabe and de Souza 2007; Shalgi et al. 2014). These analyses also found that retained introns were associated with weaker splice sites than constitutive introns. Comparison of different clusters of IR events that did not alter their PIR during erythroid differentiation showed an inverse correlation between PIR and splice site strength, consistent with a contribution of weak splice sites to IR. However, regulated events with a large dynamic range of PIR had stronger splice sites than the unregulated events, even though their maximal PIR levels were higher (Pimentel et al. 2016). Similar observations were made in smooth muscle cells (Llorian et al. 2016) and in neurons (Mauger et al. 2016). This suggests that weak splice sites within an intron definition context can predispose to IR, but are not in themselves sufficient. This is unsurprising; cassette exons also have weaker splice sites than constitutive exons (Keren et al. 2010), but are regulated in numerous distinct programs by a plethora of regulatory RNA binding proteins, by changes in the levels and activities of core splicing factors, as well as by RNA polymerase II elongation rates and chromatin contexts (Fu and Ares 2014; Naftelberg et al. 2015). It might be expected that different sets of IR events will also be co-regulated by a variety of inputs including the action of specific RBPs such as PTBP1 (Marinescu et al. 2007; Tahmasebi et al. 2016; Yap et al. 2012), hnRNPLL (Cho et al. 2014), hnRNPH, hnRNPA1, PABPN1 (Bergeron et al. 2015), Acinus (Rodor et al. 2016), and possibly G3BP (Martin et al. 2016).

As the preceding discussion has illustrated, not only can IR be regulated with different cell-type specificities, but it also encompasses a range of distinct phenomena from IR as an end-product in cytoplasmic mRNAs, to IR as a stable intermediate state in nuclear-retained RNAs awaiting the appropriate signal for completion of splicing (Boutz et al. 2015; Mauger et al. 2016; Shalgi et al. 2014), or IR as a nuclear-retained and degraded species (Yap et al. 2012). It might be expected that a range of underlying mechanisms lead to these different forms of IR, and also that the mechanism of IR might be related to the subsequent fates by, for example, influencing cytoplasmic export. IR is distinct from other types of ASE in that the IR RNA still contains a (potentially) spliceable intron. The earliest steps in spliceosome assembly are sufficient to lead to nuclear retention of an RNA (Legrain and Rosbash 1989; Takemura et al. 2011). Partial assembly of stalled or abortive splicing complexes might, therefore, be sufficient to cause nuclear retention of the IR RNA. For example, the 3′ terminal introns that are retained in response to PTBP1 in non-neuronal cells require functional splice sites to be retained in the nucleus (Yap et al. 2012). This suggests that the block to RNA export involves a splicing-related complex that has been stalled by the action of PTBP1, as has been demonstrated for PTBP1-repression of the C-SRC N1 exon, where PTBP1 stabilizes binding of U1 snRNP to a repressed 5′ splice site (Sharma et al. 2011). Whether such a stalled complex marks the transcript irreversibly for nuclear retention and decay, or whether it might subsequently disassemble as PTBP1 levels decrease during differentiation, allowing splicing to a productive mRNA, is unclear. A similar mechanism seems to operate in a homeostatic feedback loop involving PABPN1, the nuclear polyA binding protein. PABPN1 binding to its own 3′ UTR leads to IR of the 3′ terminal intron leading to nuclear retention and exosome mediated turnover (Bergeron et al. 2015). Another recently characterized IR event in *ARGLU1* coincides with an ultraconserved region and the retained intron contains a “poison” cassette exon (Pirnie et al. 2016), similar to Srsf3, 5 and 7 (Lareau et al. 2007). In this case, assembly of unproductive splicing complexes around the cassette exon appears to lead to IR and retention of the RNA in the nucleus. For the detained introns that are spliced upon Clk inhibition, it is suggested that local hyper-phosphorylation of SR proteins mediated by Clks prevents the transition from an early pre-spliceosome, where SR proteins need to be phosphorylated, to a catalytically active spliceosome (Boutz et al. 2015; Prasad et al. 1999). The paused complex would prevent nuclear export while remaining poised to respond to reductions in Clk activity, or possibly increased phosphatase activity.

In contrast to the preceding examples, IR in which splicing complexes fail to assemble, either due to very weak splice sites, or as a result of repressor mechanisms that block splicing complex assembly at the very earliest stages, would be consistent with export to the cytoplasm. The protein-coding “exitron” containing RNAs have extremely low splice site strengths and high basal PIR compared to other IR events (Braunschweig et al. 2014; Marquez et al. 2015). It seems plausible that in these cases, IR is associated with complete failure of the splicing machinery to recognize the splice sites of the retained intron, and the mRNA is then exported and translated in the same manner as any other protein-coding mRNA. Retention of the 3´ UTR intron 4 of SRSF1, which leads to avoidance of NMD (Sun et al. 2010), is promoted by binding of phosphorylated Sam68 to sites in the intron. Sam68 binding inhibits splicing, although the stage of complex assembly was not demonstrated. Nevertheless, the resultant IR RNA isoform is exported to the cytoplasm and productively translated (Valacca et al. 2010).

A number of reports support the role of chromatin and transcriptional influences on IR. Using ENCODE ChIP-Seq and matching RNA-Seq data for human K562 and mouse CH12 cells, significant enrichment of RNA Pol II was observed across retained introns compared to constitutive introns (Braunschweig et al. 2014). The enrichment was particularly marked for the large subunit C-terminal domain (CTD) hyper-phosphorylated on Serine-2 of its repeats, and for specific chromatin modifications (e.g., H3K27Ac) and chromatin proteins (e.g., CHD2). This suggests that IR is associated with accumulation of the elongating form of RNA Pol II (with S2P modified CTD). Treatment with the RNA Pol II elongation inhibitor DRB (which also inhibits serine-2 phosphorylation) also led to increased PIR of a panel of IR events. Taken together, the data indicate that pausing of RNA Pol II over retained introns correlates with increased retention, perhaps by allowing a time window within which repressive splicing regulatory complexes could become established before intron definition can take place upon synthesis of the 3´ splice site (Braunschweig et al. 2014). A possible link between DNA methylation and variation in IR was also suggested by correlations between IR and mutations in IDH1 and 2 (Dvinge and Bradley 2015). In addition, genomic loci of retained introns in differentiated breast epithelial cells possess higher CpG island density and DNA methylation compared to non-retained intronic regions (Gascard et al. 2015). Yet, another connection between IR regulation and chromatin modification was uncovered when the H3K36me3 reader BS69 was found to interact with spliceosomal components including U5snRNP protein EFTUD2 and U4snRNA (Guo et al. 2014). Specifically, BS69 upregulated IR at H3.3K36me3 regions enriched for its genomic binding in a manner that was antagonistic to EFTUD2 and dependent on SETD2, the methyl transferase responsible for laying the chromatin marks. The authors propose that BS69 promotes IR by sequestering or blocking U5 functionality while preventing U4 snRNP release from the tri-snRNP, although this remains to be largely validated (Guo et al. 2014).

A final possible contributor to regulated IR programs is alterations in the activity of the core splicing machinery. RNAi screens have shown the depletion of numerous individual core splicing factors, including components of spliceosomal snRNPs, can specifically alter cassette exon splicing patterns rather than leading to widespread failure of splicing (Papasaikas et al. 2015). IR events are also sensitive to depleted levels of core splicing factors (Braunschweig et al. 2014). Indeed, many of the developmental and stress-related programs of IR either affect core splicing components and/or are associated with observed lower levels of core splicing factors (Boutz et al. 2015; Dvinge and Bradley 2015; Edwards et al. 2016; Llorian et al. 2016; Memon et al. 2016; Pimentel et al. 2016; Shalgi et al. 2014). The extent to which IR is driven by low splicing factor activity or, conversely, IR drives lower expression of splicing factors, is currently unclear. In differentiating granulocytes, significant down-regulation of a number of U1 and U2 snRNP proteins accompanied the program of IR, although no IR or other non-productive ASEs were reported in the cognate splicing factor pre-mRNAs (Wong et al. 2013), suggesting that low splicing activity might be driving IR. This would also be consistent with the concept that an “over-stretched” splicing machinery in highly transcriptionally active cancers might lead to IR (Dvinge and Bradley 2015; Hsu et al. 2015). The weaker consensus splice sites of retained introns might be especially sensitized to reduced splicing activity. Nevertheless, it is important to note that regulation of cassette exon events is much more balanced in most regulated splicing programs with similar numbers of cassette exons being up and down-regulated at the same time that IR increases (e.g., (Llorian et al. 2016; Pimentel et al. 2014, 2016). Indeed, cassette exons that are included in differentiated smooth muscle cells, alongside a program of IR, have weaker splice sites than down-regulated cassette exons (Llorian et al. 2016), arguing that the splicing environment of differentiated quiescent cells that gives rise to increased IR is not “defective”.

## Concluding remarks

Intron retention has only recently garnered attention as a major component of the global alternative splicing-mediated regulation of cellular function. Transcriptomic profiling combined with robust quantitative analyses (Braunschweig et al. 2014; Marquez et al. 2015; Pimentel et al. 2016; Wong et al. 2013; Yap et al. 2012) has uncovered the extensive IR networks that are an integral part of many mammalian programs of gene expression. These studies have also served to underscore the functional versatility of intron retention events in regulation of mRNA expression. In addition to general NMD-induced down-regulation, IR is capable of tuning cell-type specific transcriptomes by production of diverse protein isoforms (Braunschweig et al. 2014; Marquez et al. 2015); by regulation of translation initiation via uORFs or other 5′ UTR features (Tahmasebi et al. 2016; Weatheritt et al. 2016); by regulation of mRNA stability and expression by IR in 3´ UTR (Sun et al. 2010; Thiele et al. 2006); and by rapid induction of expression via detained introns (Boutz et al. 2015; Mauger et al. 2016; Ninomiya et al. 2011).

Splice modulation therapies have recently taken centre stage in the treatment of Duchenne muscular dystrophy and spinal muscular atrophy. Antisense oligonucleotide (AON) drugs that redirect the splicing of specific cassette exons of the Dystrophin (Eteplirsen) and SMN2 (Nusinersen) genes, respectively, received FDA approval late in 2016. AONs can also be employed to modulate IR in different contexts and channel decay or protection mechanisms that enable control over mRNA and consequently protein expression. Careful design of AONs to block splice sites or mutations that induce IR (Yadegari et al. 2016) would be feasible to target specific introns (Kralovicova et al. 2014, 2016). Analysis of the mechanisms of regulation of cassette exons benefitted hugely from the ability to curate sets of tightly co-regulated events from mRNA-Seq data, followed by computational deciphering of the key features of these events, and detailed molecular dissection of individual events that exemplify the co-regulated program (Barash et al. 2010; Chen and Manley 2009; Fu and Ares 2014; Matlin et al. 2005). A similar strategy should allow a much fuller understanding of the molecular underpinnings of regulated programs of IR, and provide a better basis for therapeutic modulation of IR.
